# Editorial: Neurological, Metabolic and Inflammatory Disorders: A Common Root in Inflammasome

**DOI:** 10.3389/fphar.2021.808400

**Published:** 2021-12-07

**Authors:** Carolina Pellegrini, Luca Antonioli, Gloria Lopez-Castejon, Massimo Bertinaria

**Affiliations:** ^1^ Department of Clinical and Experimental Medicine, University of Pisa, Pisa, Italy; ^2^ Division of Infection, Immunity and Respiratory Medicine, Faculty of Biology, Medicine and Health, Lydia Becker Institute of Immunology and Inflammation, University of Manchester, Manchester Academic Health Science Centre, Manchester, United Kingdom; ^3^ Department of Drug Science and Technology, University of Turin, Torino, Italy

**Keywords:** inflammasome, neurological disorders, inflammatory diseases, phytochemicals, patients, animal models

Several lines of evidence highlight the relevance of inflammasomes in the pathophysiology of a plethora of diseases, including autoinflammatory syndromes (i.e., cryopyrin-associated autoinflammatory syndromes [CAPS]), neurological diseases, such as Parkinson’s disease (PD), Alzheimer’s disease (AD), amyotrophic lateral sclerosis (ALS) and major depressive disorder (MDD), metabolic disorders, such as obesity and type 2 diabetes, and chronic inflammatory diseases, including gout, psoriasis and intestinal inflammation (Pellegrini et al., 2019). In particular, inflammasome complexes, consisting of nucleotide-binding oligomerization domain leucine rich repeat and pyrin domain-containing protein 3 (NLRP3), NLRP6, NLRP1, NLRC4 or absent in melanoma 2 (AIM2) subunit, adaptor protein apoptosis-associated speck-like protein (ASC) and pro-caspase-1, through the processing and release of interleukin (IL)-1β and IL-18 and the induction of pyroptosis, acts as a key player both in maintaining the host physiology and in the triggering the immune/inflammatory responses in neurological, metabolic, and inflammatory diseases (Pellegrini et al., 2019). In addition, besides canonical caspase-1 dependent inflammasome signalling, non-canonical activations, which depend on caspase-11 in mice (caspase 4 and caspase 5 in humans) or caspase-8 have been found to be involved in the immune/inflammatory responses associated with such disorders (Pellegrini et al., 2019). In this setting, several studies included in our Research Topic have provided new insights on the role of inflammasome pathways in the pathophysiology of neurological and inflammatory diseases as well as on the effects of phytochemicals acting on inflammasome signalling in the counteracting central neuroinflammation and immune/inflammatory responses in such diseases. An interesting bibliometric analysis of 1,222 documents performed by Chen et al. has reported that inflammasome activation and the induction of pyroptosis in brain cells, including microglia, astrocytes, neurons, are pivotally involved in the coordinating physiological processes as well as in the promoting the pathophysiological events associated with brain disease, such as traumatic brain injuries, stroke, AD, and PD (Chen et al.). In addition, they observed that the activation of inflammasome signalling, including NLRP3, NLRC4 and NLRP3, and pyroptosis in brain disorders is associated with the increase in endoplasmic reticulum (ER) stress, oxidative stress, and mitochondrial dysfunction (Chen et al.). In support of this view, the review article by Holbrook et al. has highlighted the role of NLRP3 inflammasome in neurodegenerative diseases, including PD, AD, Huntington’s disease, ALS, and prion diseases. In particular, pooling together human and preclinical investigations, they observed that NLRP3 inflammasome overactivation and dysregulation is a common path of several neurodegenerative diseases and they suggest NLRP3 inhibition as a suitable pharmacological strategy for the management of these brain disorders (Holbrook et al.). However, Piancone et al. showed that the treatment monomeric or aggregated α-synuclein to peripheral blood mononuclear cells (PBMCs) from PD patients increased IL-1β and IL-18 as compared to PBMCs from healthy subjects, without inducing NLRP3-ASC-caspase-1 signalling activation. In addition, treatment monomeric or aggregated α-synuclein to PBMCs from PD patients was associated with an increase in IL-6 release along with a decrease in IL-10 levels, suggesting that PD-associated neuroinflammation is not the consequence of the activation of the NLRP3 inflammasome but rather of an imbalance between pro-inflammatory and anti-inflammatory cytokines. However, the authors have focused attention on NLRP3 inflammasome, leaving out the involvement of other inflammasome subtypes, including NLRP1 or NLRC4, in the triggering IL-1β, IL-18 release in PBMCs from PD patients following monomeric or aggregated α-synuclein incubation (Piancone et al.).

Of note, Song et al. proposed a novel mechanism underlying neuroinflammatory and neurodegenerative processes in PD. In particular, they showed that the increased expression of Kir6.2, regarded as an inflammatory mediator involved in degeneration of dopaminergic neurons in PD, contributed to neurotoxic astrocyte reactivity and neurodegeneration in brain tissues from mice with lipopolysaccharide-induced PD, *via* activation of dynamin-related protein 1 (Drp1)-induced mitochondrial fission (Song et al.). In this context, it is noteworthy that Drp1-induced mitochondrial fission is associated with overactivation of NLRP3 inflammasome (Pellegrini et al., 2021). Therefore, Kir6.2 could represent a potential pharmacological target aimed at counteracting astrocytic inflammation through the inhibition of mitochondrial dysfunction and likely, in turn, the blockade of inflammasome activation. However, further investigations are needed to better clarify the role of Kir6.2/Drp-1/mitochondrial dysfunction/inflammasome signalling in PD.

Besides brain disorders, an interesting article by Forouzandeh provided novel insights about the role of inflammasome signalling in psoriasis. In particular, they showed that ASC and IL-18 levels were increased in serum from patients with psoriasis as compared to controls. In addition, IL-18 levels had a statistically significant linear correlation with those of ASC, suggesting that the activation of inflammasome pathways could represent useful biomarkers for the evaluation of inflammatory state in psoriasis patients (Forouzandeh et al.).

Of interest, special attention in our Research Topic has been paid to characterize and discuss the effects of lipid mediators and phytochemicals acting on inflammasome pathways in counteracting central neuroinflammation and immune/inflammatory responses in brain and chronic inflammatory diseases. Wei et al. showed that Resolvin D1, a lipid mediator derived from docosahexaenoic acid, endowed with anti-inflammatory and neuroprotective properties, counteracted blood brain barrier (BBB) impairments and improved neurological deficits in rats with subarachnoid hemorrhage (SAH) by increasing occludin, claudin-5 and zona occludens-1 expression and decreasing nuclear factor-κB (NF-κB)p65, matrix metallopeptidase-9, intercellular cell adhesion and molecule-1 expression. The mechanisms underlying Resolvin D1 effects were ascribed to its ability to modulate inflammasome signalling, increasing A20 and inhibiting NLRP3 expression (Wei et al.). Ding et al. reported that the treatment with Evodiamine, a quinazoline alkaloid, extracted from the fruit of *Evodiae Fructus* (*Evodia rutaecarpa* Benth., Rutaceae) and endowed with antitumor, anti-nociceptive and anti-inflammatory properties, attenuated dextran sulfate sodium (DSS)-induced colitis in mice, through the inhibition of NLRP3 inflammasome complex *via* the induction of autophagosome-mediated degradation of inflammasome and the inhibition of NF-κB-mediated transcription step (Ding et al.).

Of note, in a review article, Wu et al. have highlighted and discussed current scientific evidence on the effects of phytochemicals acting at different steps of the NLRP3 inflammasome cascade in attenuating immune/inflammatory responses in lupus nephritis (Wu et al.). In particular, among phytochemicals, sophocarpine, icariin, glycyrrhizic acid, phloretin, magnolol, curcumin, and procyanidin B2 improved renal function and proteinuria and decreased serum anti-dsDNA antibody level and inflammation, by inhibiting the NF-kB pathway and decreasing NLRP3 expression (Wu et al.).

Overall, the present Research Topic provides new insights about the role of inflammasome signalling in the pathophysiology of neurological and inflammatory diseases and, most importantly, it highlights that the inhibition of inflammasome pathways could represent an innovative therapeutic strategy for the treatment of these disorders.
